# Development of fluorescence oligonucleotide probes based on cytosine- and guanine-rich sequences

**DOI:** 10.1038/s41598-020-67745-5

**Published:** 2020-07-03

**Authors:** Anna Dembska, Angelika Świtalska, Agnieszka Fedoruk-Wyszomirska, Bernard Juskowiak

**Affiliations:** 1 Faculty of Chemistry, Adam Mickiewicz University, Uniwersytetu Poznanskiego 8, 61-614 Poznan, Poland; 2 Institute of Bioorganic Chemistry, Polish Academy of Science, Noskowskiego 12/14, 60-704 Poznan, Poland

**Keywords:** Biological techniques, Biophysics, Chemistry

## Abstract

The properties of cytosine- and guanine-rich oligonucleotides contributed to employing them as sensing elements in various biosensors. In this paper, we report our current development of fluorescence oligonucleotide probes based on i-motif or G-quadruplex forming oligonucleotides for cellular measurements or bioimaging applications. Additionally, we also focus on the spectral properties of the new fluorescent silver nanoclusters based system (ChONC12-AgNCs) that is able to anchor at the Langmuir monolayer interface, which is mimicking the surface of living cells membrane.

## Introduction

Nucleic acids containing tracts of guanines (G) or cytosines (C) are often located in gene promoters and telomeres^1^ and are able to form two kinds of tetraplexes. G-quadruplex secondary structures (G4 DNA) are formed in nucleic acids by sequences that are rich in guanine (G)^2,3^; the complementary cytosine-rich DNA strand can also form a four-stranded structure called the intercalated motif (i-motif)^4,5^. G-quadruplexes can readily form in solution under physiological conditions; moreover the formation and stabilization of G-quadruplexes are dependent on monovalent cations, specifically K^+^ and Na^+^^6^. In contrast, i-motif as DNA structure containing intercalated cytosine^+^-cytosine base pairs needs to be prior protonated at N(3) of cytosine^4,5^. The properties of G-quadruplexes and i-motifs contributed to employing them as sensing element in fluorescent oligonucleotide probes/systems generating a fluorescent signal in response to changes in environmental conditions (the presence of biometals or pH changes, respectively). Thus, DNA tetraplexes belong to functional nucleic acids family as they can exhibit ligand/ion binding capacity or even enzymatic activity^7^. Especially, G-quadruplex analogues have been widely used as molecular tools for the detection of potassium ion (K^+^)^8^, whereas i-motif based nanoswitches and biosensors are suitable for monitoring pH fluctuations in the physiological range^9^. The different approaches are applied to obtain fluorescence signal upon cation- or proton-binding event, such as incorporating fluorescent nucleobases, excimer or FRET pair labelling. On the other hand, the label-free strategy for i-motif^10^ as well as G-quadruplex^11^ formation has drawn numerous attention as a simple and cost-effective alternative leading to ultrasensitive systems for bioanalytical applications^12–14^. For example, we developed fluorescent molecular beacons, which exploited (a) pyrene excimer emission, (b) FRET pair labelling, (c) 5-(1-pyrenylethynyl)-2′-deoxyuridine emission or (d) the 1,3-diazo-2-oxo-phenothiazine (analogue tC) emission as the measurable analytical signal corresponding to the proton-binding event by the pH-sensitive loop of molecular beacons^15^. In our studies upon the dual-pyrene labeled molecular beacons (MBs) with i-motif in the loop, we found that such MBs can be not only easily manipulated leading to sensors with a narrow working range and specific midpoint^16–18^; but also successfully transfected into living cells, where they accumulate in lysosomes and are able to react effectively to intracellular pH changes^16,17^. Recently, we are focused on studies upon MBs integrated with the i-motif, labeled with fluorescent cytosine analogue, tC and another fluorophore, which could act as an energy acceptor from tC^19^. Such designed MBs allow to obtain analytical signal in more long-wave range, which is always more beneficial for analysis undertaken in cellulo.

In recent years, a huge progress has been made regarding the development of new fluorescent nanomaterials such as quantum dots (QDs)^20^ nanodiamonds (NDs)^21^, carbon nanodots (C-dots)^22^, graphene oxide (GO)^23^, carbon nanotubes (CNTs)^24^, lanthanide-based upconversion nanoparticles (UCNPs)^25^, luminescent metal organic framework (MOFs)^26^, molecularly imprinted polymers (MIPs)^27^, aggregation-induced emission dots (AIE dots)^28^ or metal nanoclusters (NCs)^29^.

Among these fluorescent nanomaterials, QDs and metal nanoclusters (NCs) exhibit better photostability, tunable photoluminescence and ease of modification in comparison with organic dyes^20^. However, the metal nanoclusters generally have lower fluorescence quantum yield than QDs and organic dyes. The fluorescence properties of metal nanoclusters are correlated with the metal cluster, solvent and surface protecting ligands^29^. It is worth to mention that recently, Zhang et co-workers have analysed the origin of the photoluminescence of metal nanoclusters and proposed model based on ligand-assembly-mediated interfacial p band intermediate state (PBIS)^30^. Therefore, ligands are important in the case of metal nanoclusters as they protect them against forming larger non-emitting aggregates and oxidation. On the other hand, an aggregation-induced emission (AIE) strategy has been employed to enhance the luminescence of metal nanoclusters (NCs)^31,32^. For example, Luo et al. have engineered the surface M(I)-thiolate complexes of metal nanoclusters at the molecular level and obtained the highly luminescent metal nanoclusters with an interesting AIE emission^33^.

Compared to other ligand protected metal nanoclusters, DNA-templated metal nanoclusters exhibit interesting photophysical and chemical properties that are mainly dependent on the design of DNA templates (the base content as well as DNA form)^34^. In particular, Ag^+^ is widely used as it is able to specifically binds to DNA nucleobases^35^. In past, Raman studies demonstrated that the nitrogen of nucleobases could coordinate metal ions^36^. First, Petty and Dickson indicated that cytosine bases are the main sites for Ag atoms attachment^37^. These unique Ag^+^-DNA interactions have been exploiting by nanotechnology to generation fluorescent silver nanoclusters (AgNCs) for new applications strategies, from environmental monitoring to bioimaging and cancer therapy^29,38^. On the other hand, intensive studies led to DNA-templated silver nanoclusters with improved photophysical properties including high quantum yield, excellent brightness, photostability, and tunable emission colors from visible to near IR^34,39^. For example, Martinez et al. introduced DNA–Ag NCs aptamer that can selectively bind thrombin, and emits bright fluorescence (∼60% quantum yield) with an emission peak at ∼ 700 nm^34^. DNA-templated silver nanoclusters include both Ag^+^ ions and neutral silver atoms (Ag^0^). The first proposed model indicated that DNA–Ag NCs are composed of a neutral Ag core attached to DNA bases via peripheral Ag^+^ ions, which seem to be the essential stabilizing elements of the fluorescent clusters^40^. Later studies support the following model: the metal-like core, composed of interconnected and short Ag–Ag bonds, that weakly links to an encapsulating Ag^+^ ions—DNA shell^41^.

Silver nanoclusters stabilized with DNA scaffolds are an excellent alternative to organic fluorophores, due to bright fluorescence, ability to manipulate their emission spectrum or large Stokes shifts and the enhanced stability due to tuning the sequence of the DNA template^34,39^. Therefore, we have also started to develop fluorescent oligonucleotides probes based on silver nanoclusters. The goal of our work is to utilize short oligonucleotide (called ChONC12), rich in cytosines and equipped with a cholesterol anchor as a scaffold for the synthesis of silver nanoclusters. The ChONC12-AgNCs probe was designed and studied in order to verify its potential as a fluorescent tag that can be integrate with G-quadruplex DNA to create FRET working system able to anchor into the cellular membrane and monitor changes in potassium concentration. Previously, we proved the successful incorporation of cholesterol-modified fluorescent probes based on G-quadruplexes to the outer cell membrane of living HeLa cells^42^. So far, we have optimizied the synthesis of ChONC12-AgNCs in buffer as well as in cell lysate^19^.

In this paper, we expanded our report on current development of fluorescence oligonucleotide probes, based on i-motif or G-quadruplex forming sequences for cellular measurements^19^. As mentioned, the first part of the presented paper concentrating on studies upon MBs integrated with the i-motif, labeled with the 1,3-diazo-2-oxo-phenothiazine (cytosine fluorescent analogue, tC) and Atto520 dye at 5′ terminus. Next, we present the spectral properties of the fluorescent silver nanoclusters ChONC12-AgNCs in buffer as well as at the Langmuir monolayer interface, which mimic a biomembrane surface.

## Results and discussion

### Molecular beacons with i-motif in the loop for bioimaging applications

Molecular beacons are fluorescently labeled single-stranded oligonucleotides with a stem-loop conformation containing a single-stranded loop region that is antisense to the target sequence to be detected^43^. The way to obtain pH-sensitive molecular beacon (MB) is to enclose sequence rich in cytosines into the loop of MB^16,17^. Thus, the cytosine-rich loop of MB folds into i-motif upon changes in [H^+^], whereas the different fluorescence tags can be used to give analytical signal upon proton-binding event.

Recently, we have developed tC-MB-520 probe based on molecular beacon with i-motif in the loop, labeled with the 1,3-diazo-2-oxo-phenothiazine (cytosine fluorescent analogue, tC) and Atto520 dye at 5′ terminus (Table 1). Our design was inspired by the work of Stengel et al.^44^ using tC as a FRET-donor in a pair with Alexa-555 to study the conformational dynamics of DNA polymerase. It is worth to mention, that substitution of normal nucleobases with their fluorescent analogues is not a popular approach due to the limited number of available fluorescent nucleobases. The group of tricyclic cytosine fluorescent analogues includes 1,3-diazo-2-oxo-phenothiazine (tC), 1,3-diazo-2-oxophenoxazine (tC^O^) and 7-nitro-1,3-diaza-2-oxophenothiazine (tC_nitro_)^45^. The spectral characterization of the fluorescent probe, called tC-MB-520 was performed in the various pH solution and by using UV–Vis, CD and steady-state fluorescence spectra measurements.

**Table 1:** The studied oligodeoxyribonucleotide probes.

Name	Length	Sequence	Company
Ch(F-TBA-T)	23-mer	5′-Ch-(dT FAM)-TTT AGG TTG GTG TGG TTG GAT TT-TAMRA-3′	Eurogentec
ChONC12	15-mer	5′-Ch-CCC ACC CAC CCA CCC-3′	Eurogentec
tC-MB-520	37-mer	5′-Atto520 GTG ATC TAA CCtC CGC CCC GCC CCG CCC CTA CGA TCA C-3′	IBA
MB-520	37-mer	5′-Atto520 GTG ATC TAA CCC CGC CCC GCC CCG CCC CTA CGA TCA C-3′	IBA

**Figure 1:**
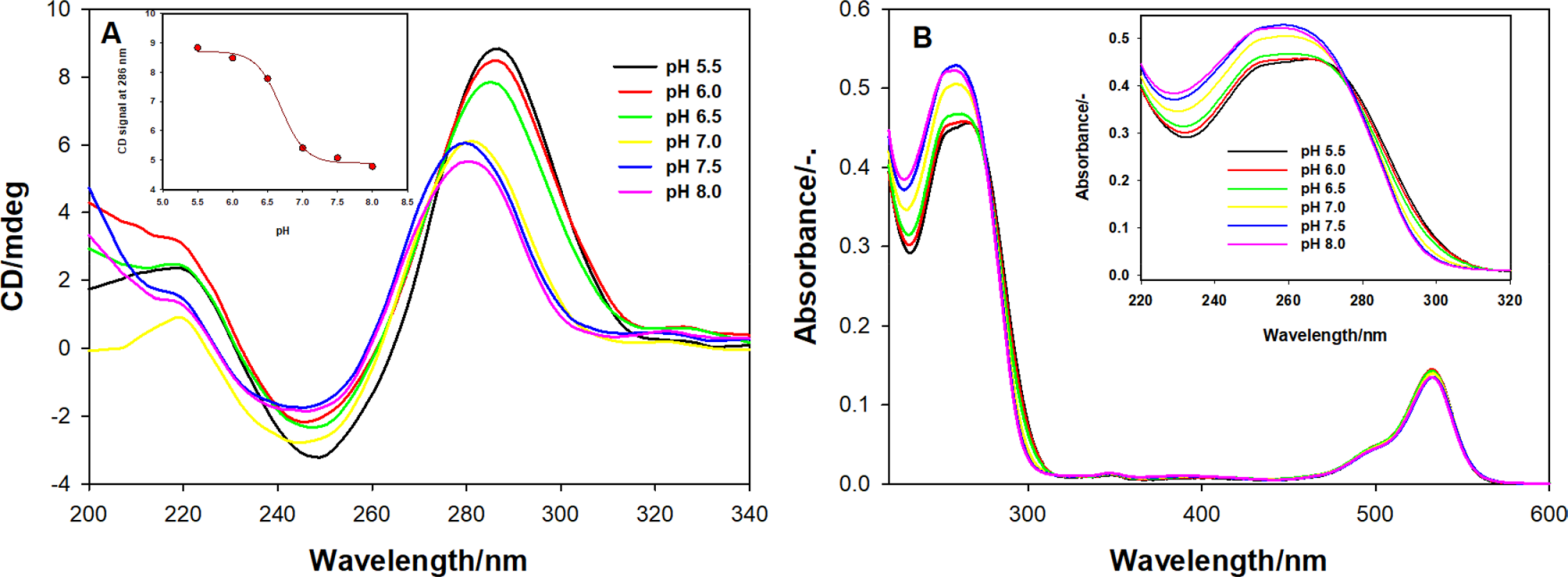
The spectra of tC-MB-520 probe (1 μM) prefolded in different pH buffers: (**A**) CD spectra with insert showing the dependence of CD signals at 286 nm against the pH values; (**B**) UV–Vis absorption spectra with insert showing absorption changes in UV region.

First, the analysis of the CD spectra confirmed that the loops of the designed molecular beacons are able to fold into i-motif. In pH range of 5.50–7.00, the CD spectra of tC-MB-520 probe exhibit a very sharp positive band at 288 nm and a weak negative band around 252 nm that is consistent with the spectral characteristics of the i-motif structure^46,47^. Furthermore, at pH values around 7.0 the positive maximum shifted toward 280 nm indicating partial unfolding of the i-motif structure (Fig. 1A). Similar spectral behaviour was observed in case of CCtC CGC CCC GCC CCG CCC CA probe^48^. For tC-MB-520 probe, CD signals observed at 286 nm were plotted against pH and fitted into a sigmoidal function. The pH of i-motif transition (pH_T_) was established at pH value 6.6 and the representative graph is shown in Fig. 1A (insert).

Another technique allowing to observe the formation of the i-motif is UV molecular absorption spectroscopy, because the protonation of cytosines induces hyperchromic effect, which can be observed at wavelengths from 275 to 300 nm, as well as the bathochromic shift associated with shifting of the absorption maximum wavelength from ~ 262 nm (for neutral cytosine) to 275 nm (for protonated cytosine)^49^. The absorption spectrum of tC-MB-520 is characterized by two clearly seen absorption bands: a wide band between 260–300 nm, mainly attributed to absorption of the nucleobases and a well-separated, low energy band > 450 nm with a local maximum at ~ 525 nm (Fig. 1B). The insert in Fig. 1B shows that i-motif formation is accompanied by decreased absorption maxima and red-shifts with increased absorption at wavelengths from 280 to 300 nm, as infered from absorbance spectra at acidic pH values. The observed changes are principally the same as evidenced for other i-motif forming oligonucleotides^50^. What is important, the protonation of 2′-deoxycytidine, which does not involve structural changes also causes a red-shift, however increased absorption maxima is observed^46^. Therefore, we proved that C-rich loop is a pH-sensing element in our molecular beacon.

**Figure 2:**
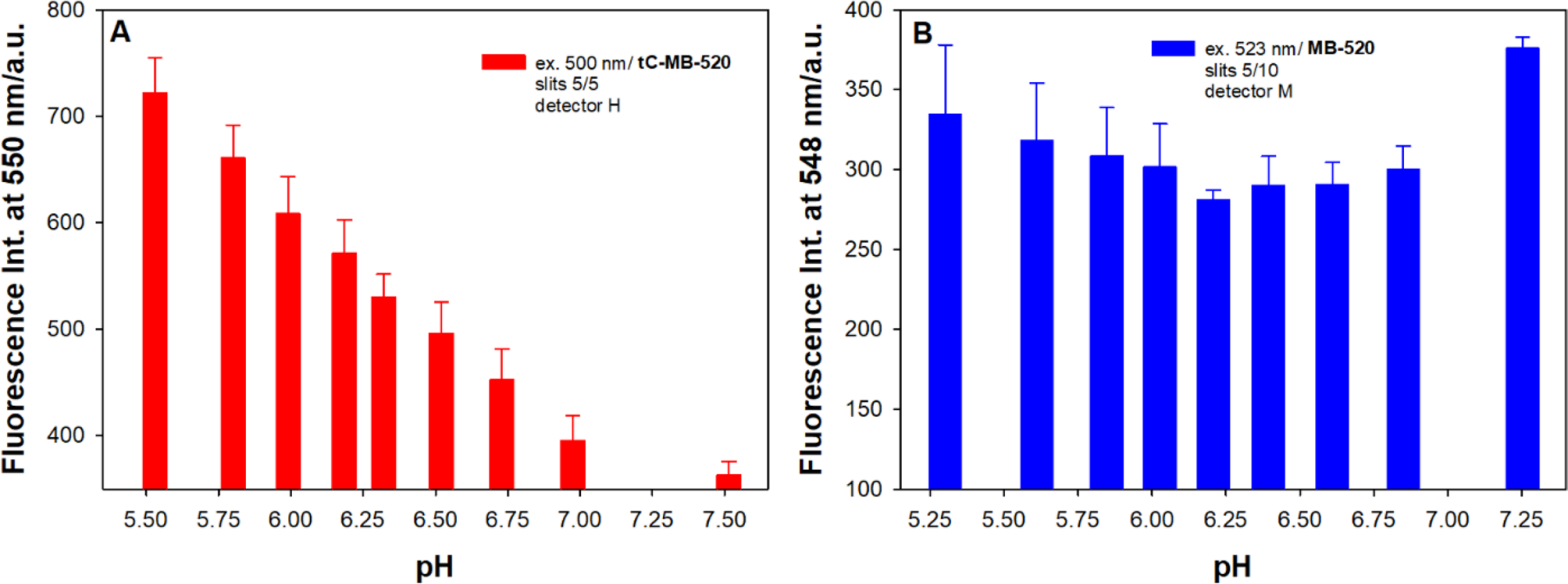
The maximum fluorescence intensity of molecular beacons in different pH solutions: (**A**) tC-MB-520 probe (0.25 μM); (**B**) MB-520 reference probe (0.25 μM).

The ability of tC-MB-520 to monitor pH changes in bulk solution was shown in Fig. 2. As reference we used MB-520 probe, labeled only with Atto520 dye at 5′ end. The fluorescence intensity of tC-MB-520 clearly increases upon addition of H^+^ (1 M HCl, 1 μL) into buffer solution, whereas emission of MB-520 remains almost at the same level. These results indicate that tC fluorophore is essential for receiving fluorescence signal of Atto520 dependent on pH changes. Previously, we have indicated that emission of molecular beacon containing tC analogue incorporated in cytosine-rich loop underwent gradual quenching upon changing pH from 8.0 to 5.5^15^. This efficiently quenched fluorescence of tC was ascribed to the protonated tC-H^+^ fluorophore that was generated upon i-motif formation^48,51^. Thus, we expected that tC-MB-520 fluorescence signal would rather decrease upon pH lowering instead of increasing as shown in Fig. 2A. The complex fluorescence studies are needed to understand the mechanism of observed enhancement of Atto520 emission in the presence of tC-H^+^ fluorophore.

**Figure 3:**
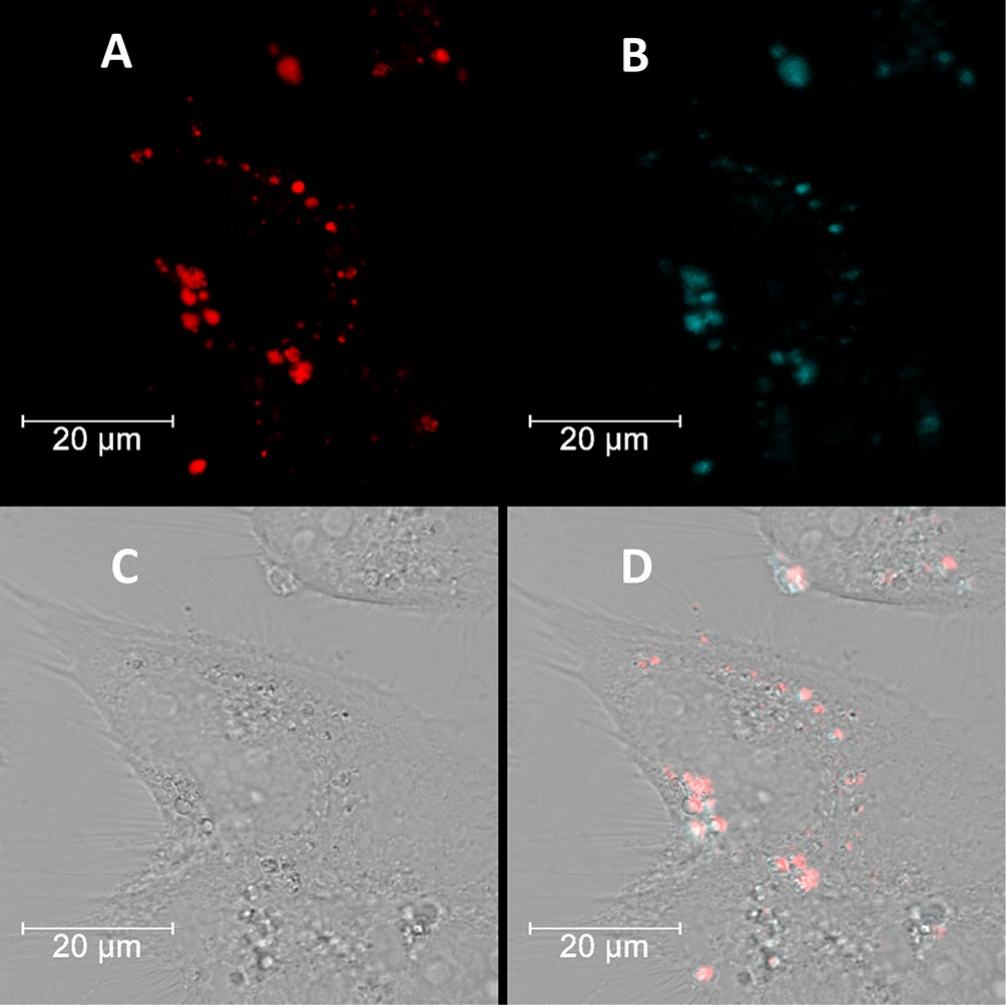
Confocal microscopy images of HeLa cells transfected with 50 nM tC-MB-520 using Lipofectamine 2000: (**A**) Atto520 fluorescence, marked red; (**B**) tC/Atto520 fluorescence, marked blue (**C**) bright field image (**D**) overlay of all images. Fluorescence emission filters: (1) for red color, 520–640 nm; excitation wavelength: 514 nm; (2) for blue color, 520–640 nm; excitation wavelength: 405 nm. Scale bars: 20 μm.

Anyway, we decided to test capacity of tC-MB-520 probe to sense pH change in living cells. In our preliminary studies, tC-MB-520 probe was transfected into HeLa cells and the confocal fluorescence imaging of HeLa cells was performed. As shown in Fig. 3, it can be clearly observed that the fluorescence signal of Atto520 is distributed as the dots collected in specific organelles, probably in lysosomes. The obtained control images indicated that the dot-shaped fluorescence signal was observed only in the HeLa cells treated with tC-MB-520. The further experiments verifying the usefulness of tC-MB-520 probe for the quantitative pH analysis *in cellulo* are planned.

### G-quadruplex interaction with cell membrane

**Figure 4:**
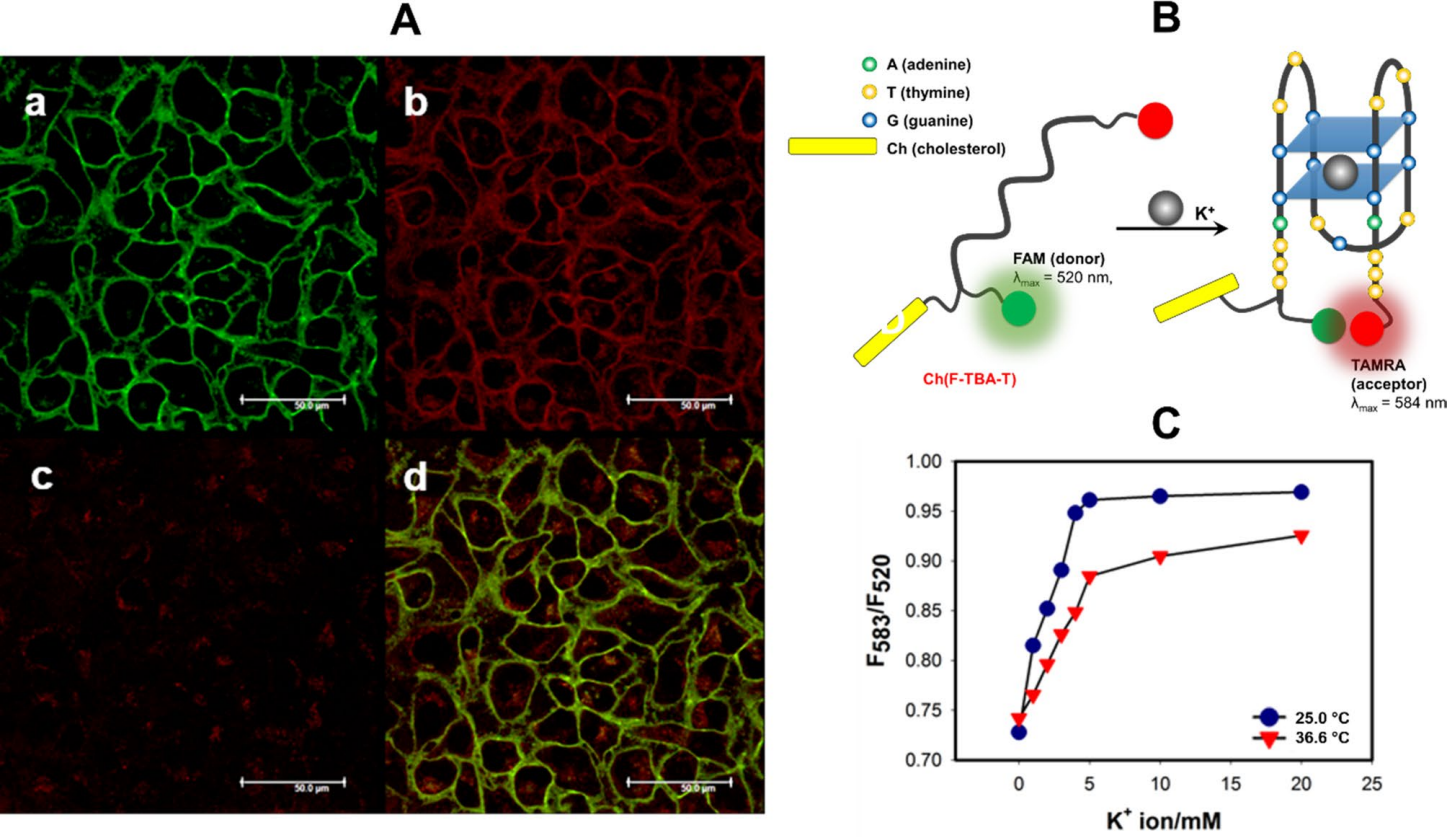
Confocal microscopy images of HeLa cells treated with 50 nM Ch(F-TBA-T) for 3.5 h (**A**): (a) FAM fluorescence, marked green; (b) FRET image, marked red; (c) TAMRA image, marked red; (d) overlay of all images. Fluorescence emission filters: (1) for FAM, 510–540 nm; excitation wavelength: 480 nm; (2) for FRET, 595–630 nm; excitation wavelength: 480 nm; (3) for TAMRA, 595–630 nm; excitation wavelength: 560 nm. Scale bars: 50 μm. (**B**) Scheme of using the FRET process to generate a fluorescent signal by the Ch(F-TBA-T) probe; (**C**) fluorescence intensity ratio (F_583_/F_520_) for Ch(F-TBA-T) (**C**) plotted against K^+^ concentration in the presence 150 mM Na^+^ at 25.0 °C (circles) and 36.6 °C (triangles).

The ability of cholesterol-modified ssDNA for spontaneous anchoring into the hydrophobic interior of lipid membranes was proven by Patolsky et al.^52^. In our group, we developed cholesterol-anchored fluorescent probes based on G-quadruplexes for spontaneous anchoring into the hydrophobic interior of living cell membrane^42^. The Ch(F-TBA-T) probe was labeled with carboxyfluorescein (FAM) and carboxytetramethylrhodamine (TAMRA) dyes (Table 1, Fig. 4B) and showed a very high binding preference for K^+^ over Na^+^ ions. Precisely speaking, the Ch(F-TBA-T) probe showed a dynamic range for K^+^ detection of 2–10 mM in the presence of 150 mM Na^+^ (Fig. 4C), which is a clinically important concentration range of K^+^ under an extracellular conditions. Fluorescent bioimages indicated the spontaneous anchoring of the Ch(F-TBA-T) to the outer cell membrane of HeLa cells within 0.5 h after adding probe into medium. The longer contact of Hela cells with Ch(F-TBA-T) probe (> 2 h) resulted in diffusion of the probe into the nucleus of the cells (Fig. 4A). What is important, the analogous probe without cholesterol moiety was not only unable to anchor to the cell membrane, but also did not penetrate inside the cells^42^. These results revealed the important role of the cholesterol group in the localization of G-quadruplex probes on the cell membrane.

The successful results of the bioimaging experiments with the cholesterol-bearing fluorescent G-quadruplex probe encouraged us to develop a new sensing system by using silver nanoclusters as a fluorescent tag.

### DNA-templated silver nanoclusters with cholesterol moiety

The DNA template used for synthesis of silver nanoclusters is 15-mer oligonucleotide with cytosine-rich segments 5′-CCCACCCACCCACCC-3′. Ag^+^ possesses a preferred high affinity to cytosine base (C) over adenine (A), guanine (G) and thymine (T) bases as indicated by ^1^H NMR spectra and DFT results^53^. Moreover, a binding constant of the C–Ag^+^–C base pair is comparable to that of the T–Hg^2+^–T base pair^54^. Additionally, the cholesterol moiety is attached to the 5′ terminus of the studied oligonucleotide. The cholesterol modification should promote the incorporation of the ChONC12 to the lipid membranes and further to the cellular membrane.

We synthesized the ChONC12-AgNCs nanoclusters by adding sodium borohydride to a buffer solution of silver nitrate and the ChONC12 oligonucleotide under air, as described in the literature^55^. The formation of the nanoclusters during the reduction step was evident from the appearance of a yellow color. Absorbance, CD and fluorescence spectra characterized the nanoclusters that formed following the reduction of the DNA-bound silver cations.

**Figure 5:**
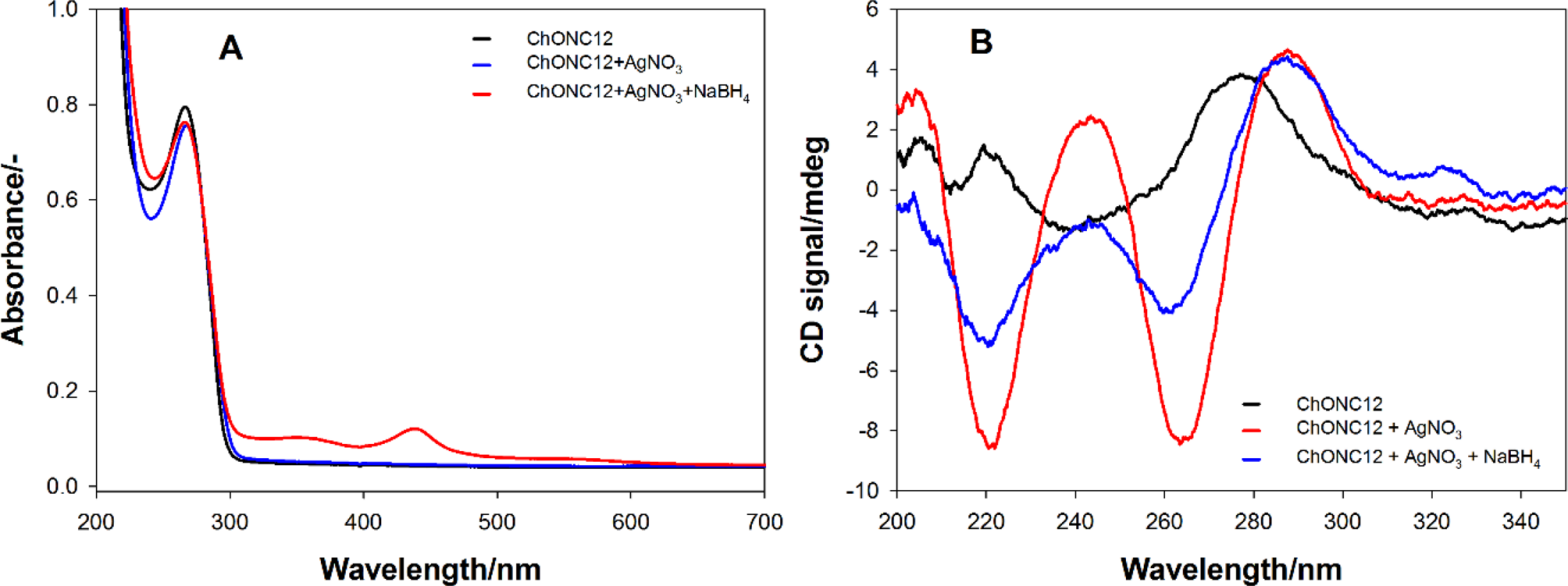
Absorption spectra (**A**) and CD spectra (**B**) of the ChONC12-AgNCs. The solutions contained 2 µM ChONC12, Tris–CH_3_COOH buffer (10 mM, pH = 7.5), [Ag^+^] = [BH_4_] = 24 µM.

Figure 5A shows the UV/Vis spectra of ChONC12 oligonucleotides before and after the addition of AgNO_3_ and NaBH_4_. In the case of the short-wave spectrum range, the main chromophores in the DNA are nucleobases, whose absorption bands are in the range below 300 nm, with maxima of approx. 260 nm. Several electronic transitions are also observed in the long-term range of the absorption spectrum of ChONC12-AgNCs nanoclusters. A primary distinguishing feature of the nanoclusters is their strong peak at 440 nm that is red-shifted and narrower relative to the plasmon transition of the nanoparticles^55^. In addition, bands at 350 nm and small at 550 nm are observed for the ChONC12-AgNCs nanoclusters. These bands are all considered as indicative of genuine nanoclusters because they are absent in the absorption spectrum of silver nanoparticles, which only displays one broad peak at λ_max_ = 405 nm^55^.

To provide more insight into the formation of the silver nanoclusters, CD spectra were recorded. In the absence of Ag^+^, the ChONC12 solution (black line, Fig. 5B) shows the expected positive peak around 280 nm coming from predominantly unstructured cytosine oligonucleotides^46,56^. Ag^+^ binds preferentially with the DNA bases, and the circular dichroism spectrum shows the significant effect of Ag^+^ on the DNA conformation, as two strong peaks with negative ellipticity develop at 220 nm and 265 nm and positive peak shifted to 290 nm (red line, Fig. 5B). These results are consistent with reported studies of the effect of Ag^+^ on polynucleotides containing of uridine and inosine^57^. The reduction with BH_4_^-^ Ag^+^ ions only gives a decrease in the intensity of negative bands (220 nm and 265 nm) (blue line, Fig. 5B). These elliptical changes suggest that nanoclusters retain the chirality of the DNA template.

**Figure 6:**
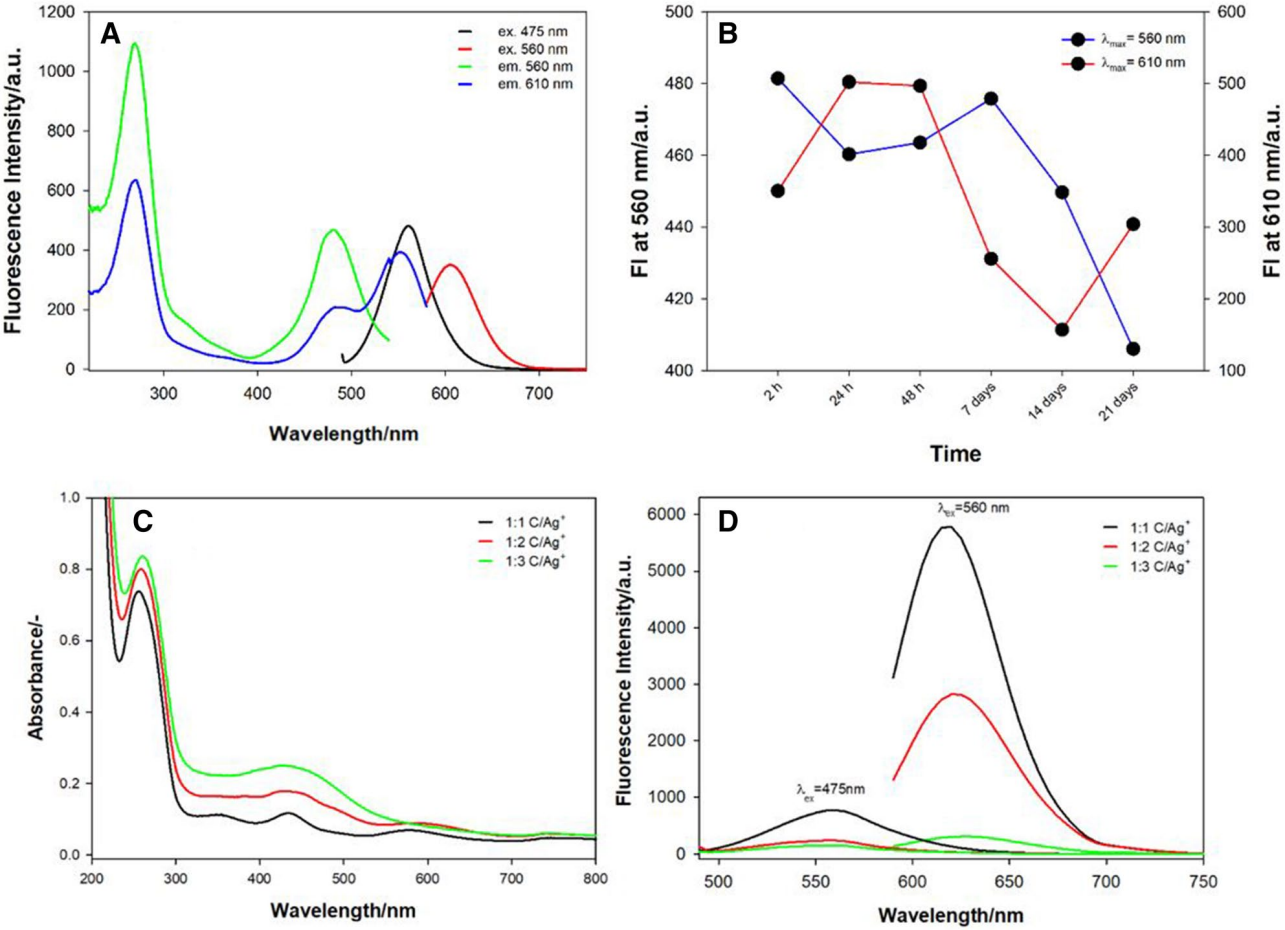
*Upper panel*: Excitation and emission spectra of ChONC12Ag-NCs (**A**) and the effect of time on the emission stability of ChONC12-AgNCs for 1 h to 21 days after the reduction of silver (**B**). Conditions: 2 µM DNA, Tris–CH_3_COOH buffer solution (10 mM, pH 7.5), [Ag^+^] = [BH_4_^−^] = 24 µM; λ_ex_ = 475 nm/λ_em_ = 560 nm, λ_ex_ = 560 nm/λ_em_ = 610 nm. *Lower panel*: Absorbance (**C**) and emission (**D**) spectra of ChONC12-AgNCs nanoclusters obtained at different molar ratio C/Ag^+^. Conditions: 2 μM DNA, 10 mM Tris-CH3COOH (pH = 7.5), λ_ex_ = 475 nm and λ_ex_ = 560 nm.

A characteristic feature of the silver nanoclusters, unlike most metal nanoparticles, is their strong fluorescence due to the lower density of electronic states. The sequences and lengths of the template DNA strands play significant roles in determining the sizes of the DNA-AgNCs and thus, their optical properties; in contrast, the Ag^+^/DNA molar ratio determines the fluorescence intensity^58–60^. The three different Ag^+^/DNA molar ratios were tested for preparation of the ChONC12-AgNCs nanoclusters. The ChONC12-AgNCs were prepared at 1:1, 2:1 and 3:1 molar ratios of Ag^+^/cytosine bases (C) (Fig. 6, *lower panel*). As expected, the Ag^+^/C molar ratio influenced on ChONC12-AgNCs fluorescence intensity without shifting the emission wavelengths (λmax = 560 nm and λmax = 610 nm). Moreover, the ChONC12-AgNCs prepared at a 1:1 Ag^+^/C molar ratio showed the highest fluorescence intensity values (at both emission wavelengths), whereas their emission is 50% or 95% quenched as prepared at 2:1 or 3:1 ratio (Ag^+^/C), respectively. Fluorescence spectra for the as-prepared ChONC12-AgNCs are shown in Fig. 6A. After the addition of NaBH_4_ to the Ag^+^-C12 complex, the high reduction capacity of the solution promotes the formation of reduced silver nanoclusters with two electronic transitions at λ_ex_ = 475 nm/λ_em_ = 560 nm and λ_ex_ = 560 nm/λ_em_ = 610 nm. These peaks are distinguished into two types based on the highest fluorescence values and showed their evolution with time (Fig. 6B). The intensities of the yellow emission band (blue line, Fig. 6B) is decreasing with time. In contrast, the intensities of the red emission band (red line, Fig. 6B) increases (2–48 h) and then decreases with time after ≈ 48 h. Two bands with different spectral parameters: yellow one (λ_max_ = 560 nm) and red one (λ_max_ = 620 nm) emission indicate the presence of nanoclusters with different sizes. These transitions are in the spectral region for small silver nanoclusters, as expected from theoretical and experimental studies^55,61–63^. Especially, it has been proved that four neutral atoms produce green fluorescence and six neutral Ag atoms produce red fluorescence regardless of the number of Ag^+^^64^. The main purpose of the study is to introduce silver nanoclusters into cells, therefore the initial synthesis of NCs in diluted cell lysate (1%, v/v) was performed^19^. The obtained absorption and fluorescence spectra confirmed successful formation of the nanocluster structure in such a solution.

### Film balance and fluorescence studies of ChONC12–AgNCs at the monolayer interface

**Scheme 1:**
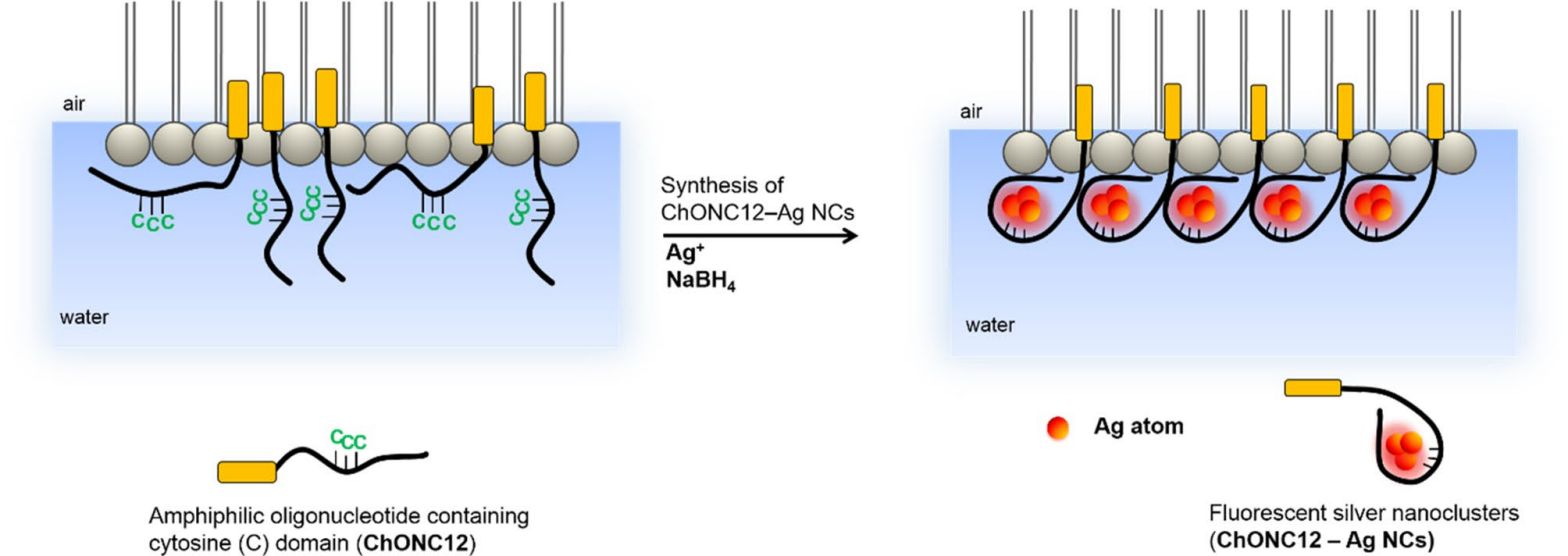
Scheme showing the synthesis of ChONC12-AgNCs incorporated into the Langmuir monolayer.

The next step involved the incorporation of amphiphilic DNA–templated silver nanoclusters (ChONC12–AgNCs) into the Langmuir monolayer at the air/water interface. Cationic monolayer of dioctadecyldimethylammonium bromide (DODAB) has been used as a model of biological membrane. Cholesterol-modified C-rich DNA (ChONC12) used in this study, is expected to undergo spontaneous anchoring into the hydrophobic interior of lipid membranes. Chemically modified DNA molecules with lipophilic group have been shown to possess high affinity for the lipid membrane. The advantages of using cholesterol include fast anchoring of DNA (few minutes) and compatibility of an anchor with a naturally occurring membrane (as cholesterol is a constituent of cell membrane (35–45%). Such an approach eliminates the risk of side effects induced by chemically reactive lipid head groups of incorporated artificial membrane constituents and this hydrophobic anchor enables a practically irreversible coupling of the oligonucleotide to the membrane^52,65,66^. Scheme 1 shows the concept of silver nanocluster synthesis on the Langmuir monolayer.

**Figure 7:**
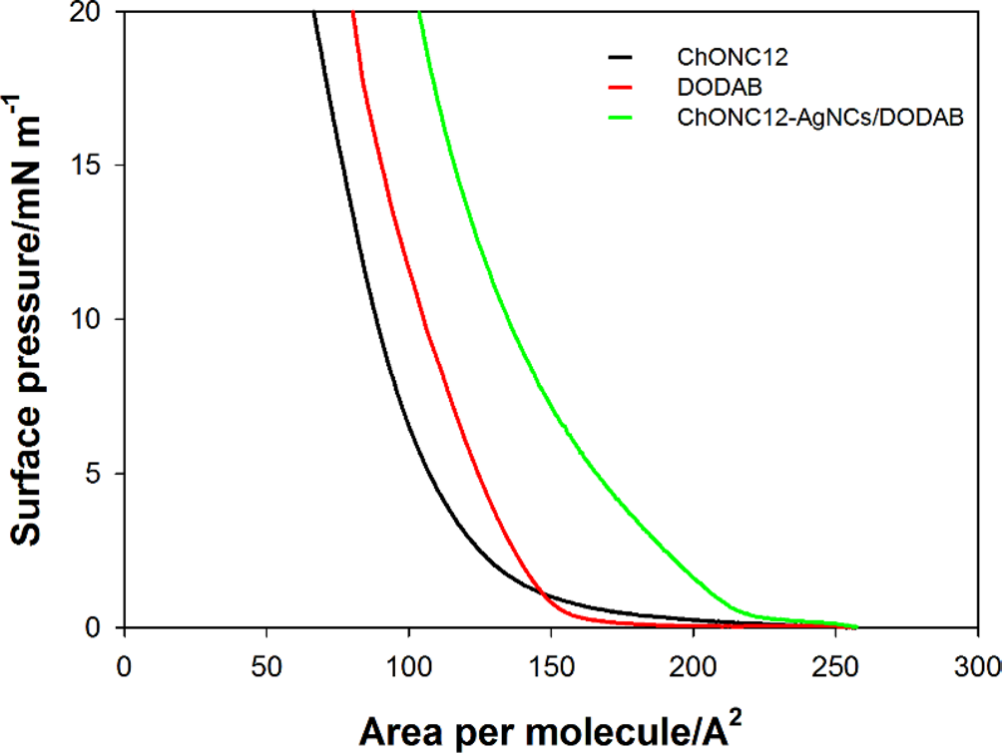
The π-A isotherms recorded for ChONC12 (black line), DODAB monolayer (red line) and ChONC12-AgNCs/DODAB complex (green line). Conditions: 50 µl (1 × 10^–4^ M) ChONC12, 5 µl DODAB (1 × 10^–3^ M), [Ag^+^] = 6 µl (1 × 10^–2^ M), [BH_4_^-^] = 120 µl (1 × 10^–2^ M); subphase contained 10 mM buffer Tris–CH_3_COOH (pH = 7.5).

The cholesterol-linked oligonucleotide (ChONC12) was soluble in all physiologically relevant solutions and could interact with the Langmuir monolayer via hydrophobic interaction between the cholesterol moiety and the membrane^42,67^, or via electrostatic interaction between the positively charged DODAB head groups and the negatively charged phosphate groups of DNA^68–70^. Figure 7 shows π-A isotherms recorded for ChONC12 oligonucleotide (black line), the DODAB monolayer (red line) and ChONC12-AgNCs/DODAB complex (green line) on the subphase containing 10 mM Tris-CH_3_COOH buffer. It can be seen that the presence of DNA strand equipped with a cholesterol anchor generates a monolayer with a surface area per molecule of about 140 Å^2^ (black line, Fig. 7). This agrees with reports that cholesterol self-assembles as a monolayer at the air/water interface and, upon compression, forms an ordered monolayer film of trigonal symmetry *P3* but with low lateral order^71^. In contrast, isotherm recorded for ChONC12-AgNCs/DODAB monolayers are shifted into the higher surface area per molecule (218 Å^2^) in relation to the ChONC12 as well as to the DODAB isotherm (154 Å^2^) (red line, Fig. 7). In this case, besides electrostatic interaction between the positively charged DODAB monolayer and the negatively charged DNA phosphates, the hydrophobic insertion of cholesterol moieties into the monolayer should be considered. However, in the case of the influence of silver nanoclusters, it can be presumed that the formation of the ChONC12/Ag^+^/DODAB complex causes strong repulsive interactions between the positively charged monolayer (positive frontal groups of DODAB) and the Ag^+^/DNA complex with the same charge, while the reduction of BH_4_^-^ creates a more compact system.

The fluorescence spectra of the ChONC12-AgNCs/DODAB system at the lipid monolayer/subphase were recorded using a fiber optics accessory interfaced with spectrofluorometer. The fiber-optic unit allows for the registration of in situ fluorescence spectra at the surface monolayer at varying surface pressure. The excitation wavelength (λ_ex_) was set at 460 nm and 550 nm (λ_em_ = 550 nm/λ_em_ = 610 nm) in order to minimize scattering at the spectral range of interest. The background scattering spectrum of the buffer subphase was subtracted from each spectrum of AgNCs/DODAB complex.

**Figure 8:**
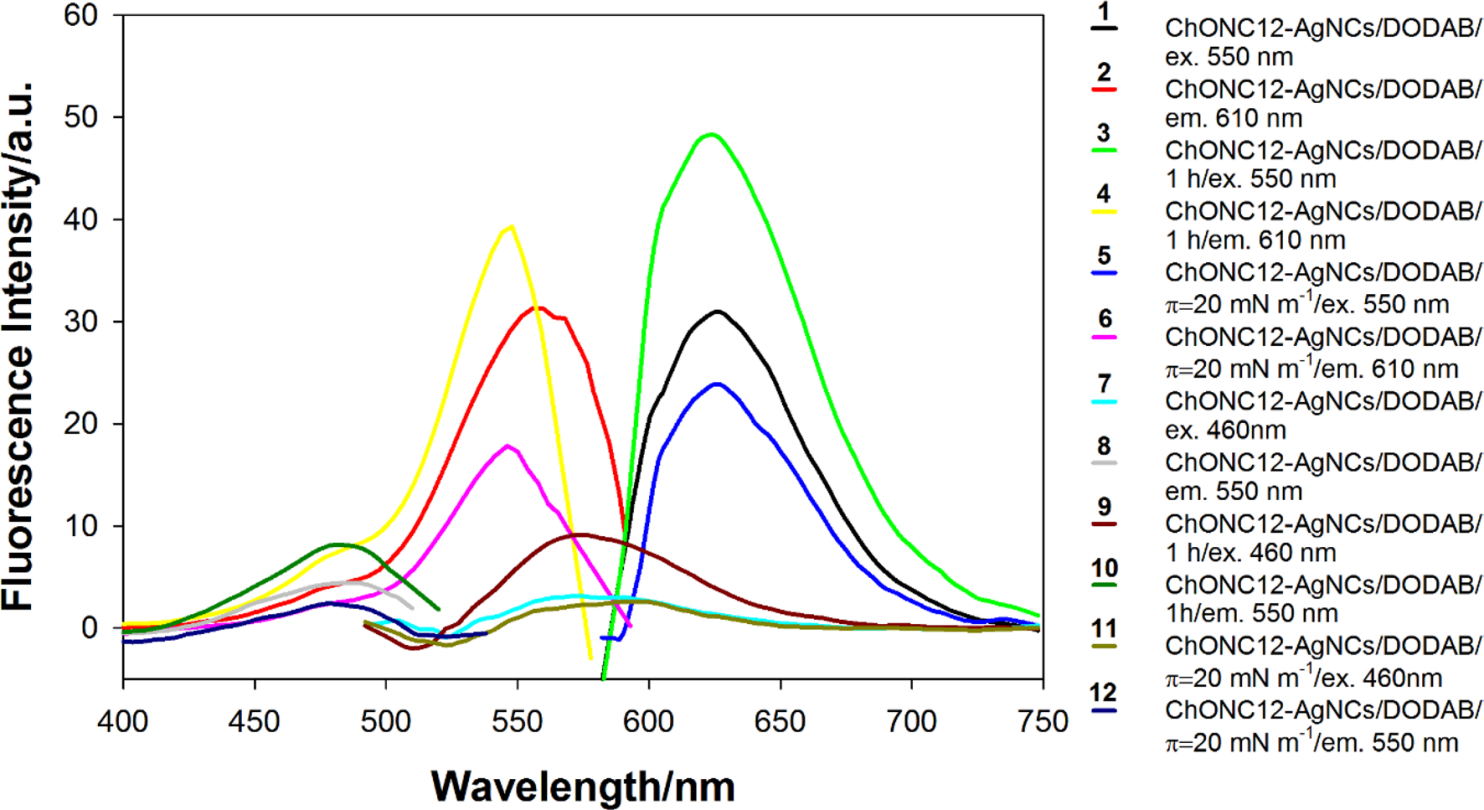
Excitation and emission spectra of ChONC12-AgNCs/DODAB system recorded at the air/water interface. Conditions: 50 µl (1 × 10^–4^ M) ChONC12, 5 µl DODAB (1 × 10^–3^ M), [Ag^+^] = 6 µl (1 × 10^–2^ M), [BH_4_^−^] = 120 µl (1 × 10^–2^ M); subphase contained 10 mM buffer Tris-CH_3_COOH (pH = 7.5), λ_ex_ = 475 nm/λ_em_ = 560 nm, λ_ex_ = 560 nm/λ_em_ = 620 nm.

The synthesis of nanoclusters directly on the Langmuir monolayer was carried out using special experimental procedure. For this purpose, a DNA template solution (ChONC12) with AgNO_3_ was prepared in water, and after 1 h was added to the chloroform solution of DODAB and the resulting mixture was applied to the subphase. Freshly prepared reducing solution of sodium borohydride (NaBH_4_) was added to the subphase and fluorescence spectra were recorded as described in the legend in Fig. 8. Excitation at 460 nm and 550 nm generated different emission spectra. Immediately after the addition of NaBH_4_ (after about 5 min), the respective emission bands appeared with maxima at 560 nm and 620 nm, indicating the formation of silver nanoclusters. Increasing the borohydride diffusion and reaction time to one hour enhanced both fluorescence bands: at λ_max_ = 560 nm by about 200% (brown line, Fig. 8) and at λ_max_ = 620 nm by about 60% (green line, Fig. 8), but compressing the monolayer to 20 mN/m caused a decrease in intensity in both cases (lines 5 and 11, Fig. 8).

Concluding, the ChONC12-AgNCs system exhibits fluorescence in the near-IR region, thereby providing optical transparency for excitation and lower background fluorescence when analyzing biological samples. Additionally, the construction of the sensor phase consisting in the incorporation of fluorescent DNA/silver nanoclusters into the Langmuir monolayer and deposition of this film onto a solid carrier should result in the development of new sensing system, in which the AgNCs analytical signal will be effectively modulated by the interaction with specific bioanalytes.

### DNA/silver nanoclusters interaction with cell membrane

**Figure 9:**
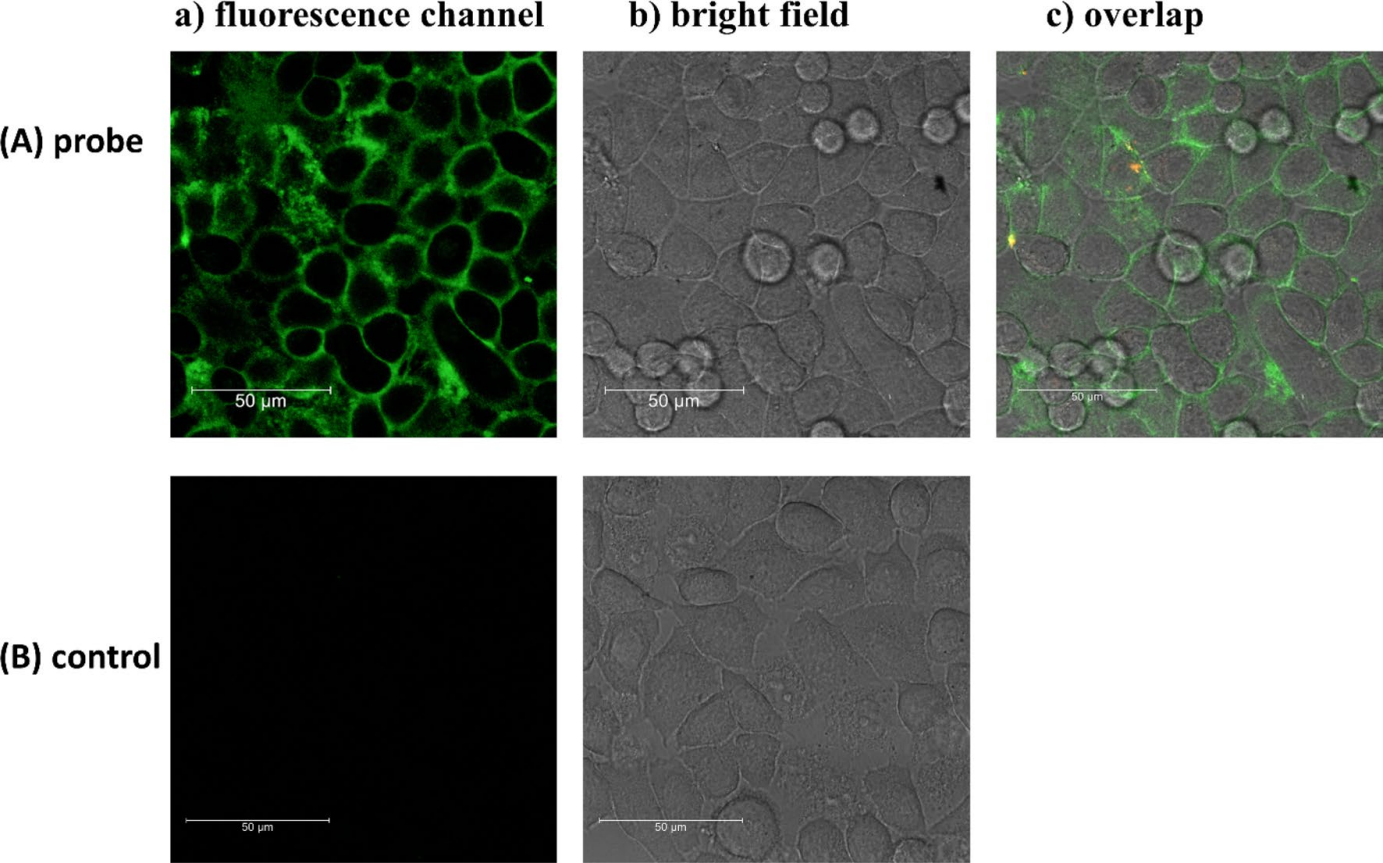
Confocal fluorescence imaging of HeLa cells loaded with 1 μM ChONC12-AgNCs for 2 h (**A**) or control HeLa cells treated with AgNO_3_ for 30 min (**B**). Fluorescence emission filter: 510–550 nm; excitation wavelength: 500 nm. Scale bars: 50 μm.

Encouraged by the outstanding interaction of the Ch(F-TBA-T) probe with cell membrane mentioned above, we decided to test cell membrane-anchoring ability of the ChONC12-AgNCs system. After incubation of the HeLa cells with 250 nM ChONC12-AgNCs for 30 min or longer (up to 2 h), the fluorescence in the cell membrane was not observed. For a good imaging performance, the concentration of ChONC12-AgNCs used in this experiment was established as high as 1 µM and excitation wavelength was set at 500 nm. As shown in Fig. 9, the cell patterns fluorescence channel overlapped well with those from the bright field image, which demonstrated that ChONC12-AgNCs can anchor onto the cell membrane. It should be noted that the control HeLa cells treated with AgNO_3_ (15 μl, 10 mM), did not produce fluorescence signals during reference experiment. These results confirmed the important role of the cholesterol-functionalized DNA template in forming of silver nanoclusters and then assisting in the localization of the probes on the cell membrane.

The obtained results clearly indicate the potential usage of ChONC12-AgNCs as element of nanosensors dedicated to monitor and visualize ions transmembrane transport.

## Conclusions

We have demonstrated that molecular beacon, tC-MB-520 integrated with the i-motif, labeled with fluorescent cytosine analogue (tC) and Atto520 dye at 5′-end, can be used to monitor pH changes in the range from 5.5 pH to 7.0. Moreover, tC-MB-520 probe could be successfully transfected into living cells.

The structurally simple cholesterol-based fluorescent G-quadruplexes (Ch(F-TBA-T)) exhibited good potential for in vivo monitoring the gradient of K^+^ ions near the cell membrane. We also employed DNA strand rich in cytosine bases (ChONC12) equipped with a cholesterol anchor as a scaffold for the synthesis of silver nanoclusters. We have tested behaviour of ChONC12-AgNCs in aqueous solution and at lipid monolayer. The obtained highly fluorescent ChONC12-Ag nanoclusters are also planned to be integrated with a G-quadruplex DNA to form the new sensing probes, which could allow visualization of transmembrane transport of cations such as K^+^ or Na^+^ by means of fluorescence microscopy. So far, the use of confocal fluorescence microscopy indicated successful anchoring of the cholesterol-bearing fluorescent ChONC12-AgNCs to the living cell membrane. However, additional work is needed to optimize the properties of DNA-templated AgNCs in cellular conditions prior to their use as part of nanosenors for quantitative bioimaging applications.

## Materials and methods

The oligodeoxyribonucleotides used in this study were synthesized by commercial companies as indicated in Table 1. All oligonucleotides were purified by reversed phase HPLC and their identities were confirmed by MALDI-TOF MS. All other reagents were purchased from Sigma Aldrich (St. Louis, MO, USA) and were used as received. Milli-Q ultrapure water was used in all experiments.

Except for microscope images, the figures were created using SigmaPlot 13.0 (Systat Software, Inc., San Jose, CA, United States).

### Spectral characterization of tC-MB-520 and MB-520 probes

#### UV/Vis and circular dichroism (CD) spectra of tC-MB-520 and MB-520 probes

Absorption and CD spectra were recorded in 1 cm path length quartz cells using a Cary 100 UV–Vis spectrophotometer (Agilent Technologies, Australia) and a Jasco J-820 Spectropolarimeter (Jasco, Tokyo, Japan), respectively. Both apparatus were equipped with a PTC-423L temperature controller to maintain 25 °C during measurements. Each CD spectra is the average of three scans from 340 to 200 nm, with a scan rate of 200 nm/min.

#### Fluorescence spectra of tC-MB-520 and MB-520 probes

Fluorescence measurements were performed on a Cary Eclipse spectrofluorimeter (Agilent Technologies, Australia) and were carried out using 0.4 × 1 cm quartz cuvettes containing 1 ml of 250 nM probe solution, prefolded in 10 mM cacodylic buffer, pH 7.50. Emission spectra of tC-MB-520 were recorded in the 510–750 nm range with λ_ex_ = 500 nm (slits 5/5 nm, detector high). Emission spectra of MB-520 were collected from 420 to 700 nm λ_ex_ = 523 nm (slits 5/10 nm, detector medium). The fluorescence spectra were recorded after addition of 1 M HCl (1 μL additions). Mean of fluorescence signal at 550 nm (or 548 nm) from at least two independent experiments and their SD were plotted for each pH value.

### Cell culturing

HeLa cells culturing was performed as we have done previously^17,42^. Thus, HeLa cells were seeded at a density of 1.2 × 10^5^ cells per well in 4-chamber glass-bottom cell culture dishes (Grenier Bio-One, Kremsmünster, Austria) and cultured in RPMI 1,640 medium (Sigma) supplemented with 10% (v/v) fetal bovine serum (FBS) (Gibco), and 1% RPMI 1,640 vitamin solution (Sigma) and 1% antibiotic antimycotic solution (Sigma) at 37 °C under a 5% CO_2_ atmosphere. After one day, the cells reached the appropriate density (80–90% confluence) and were placed in fresh RPMI 1,640 medium without supplements. Then the tC-MB-520 probe was transfected at 250 nM concentration using Lipofectamine 2000 (2 μl per well) overnight. The ChONC12-AgNCs probe was transfected in the final concentration of 1 µM for 2 h. The visualizations for tC-MB-520 and ChONC12-AgNCs were done after 24 h and 2 h of treatment, respectively. The three other sample sets with Ch(F-TBA-T) (50 nM) were prepared 3.5 h, 2.0 h and 30 min before visualization. The negative control was untreated cells. Before fluorescence confocal microscopy analysis, cells were washed twice with phosphate buffered saline (PBS) and placed in FluoroBright Live Cell Fluorescence Imaging Medium.

### Fluorescence imaging experiments

Live cell imaging was performed using a Leica TCS SP5 II confocal laser scanning microscope with a Plan Apo 63 × 1.4 NA oil-immersion objective and an environmental cell culture chamber as in our previous work^17,42^.

The fluorescence imaging was taken using excitation/emission (ex/em) wavelengths as follows:

- Atto520 channel, ex/em range = 514/520–640 nm.
- tC/Atto520 channel, ex/em range = 405/520–640 nm.
- FAM channel, ex/em range = 480/510–540 nm.
- TAMRA channel, ex/em range = 560/595–630 nm.
- FRET channel, ex/em range = 480/595–630 nm.
- Fluorescence channel for DNA-AgNCs, ex/em range = 500/510–550 nm.

Leica LAS AF 2.7.3 and Leica LAS X 3.3.3 software with a 3D deconvolution module were used for image processing and fluorescence analysis, respectively.

### Synthesis and spectral characterization of DNA-AgNCs

#### Synthesis of fluorescent DNA–AgNCs

**Scheme 2:**
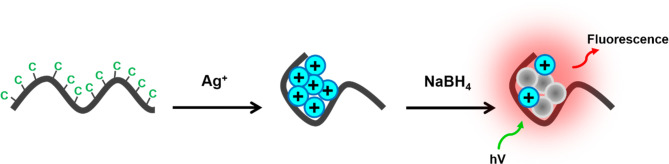
Schematic diagram of the preparation of DNA-Ag NCs.

Silver nanoclusters were synthesized following the procedure of Ritchie et al.^55^. First, the corresponding DNA solution of 2.0 μM was prepared in advance in 10 mM Tris-CH_3_COOH at pH 7.5. Then, the appropriate volume of AgNO_3_ solution of 10 mM was added into the prepared corresponding DNA solution (1:1, Ag^+^/C base, molar ratio). Next, after incubating at 4 °C for 60 min, the freshly prepared NaBH_4_ solution was added to the above mixture and shaken vigorously for 1 min (1:1, Ag^+^/NaBH_4_, molar ratio) to reduce silver ions and to form AgNCs on DNA. Finally, the obtained solution was further stored in the dark at 4 °C for 2 h to prepare stable DNA-AgNCs. The schematic diagram of the preparation of DNA-AgNCs is presented on Scheme 2.

Spectroscopy measurements of fluorescent DNA-AgNCs were performed at 25 °C using 2 µM solutions of a ChONC12 oligonucleotide in 10 mM Tris–CH_3_COOH buffer (pH 7.5) as follows.

#### Absorption spectra of fluorescent DNA–AgNCs

UV–Vis spectra of the DNA-AgNCs were recorded in the spectral range of 200–800 nm by means of Jasco V-750 spectrophotometer (Jasco, Tokyo, Japan).

#### CD spectra of fluorescent DNA–AgNCs

CD measurements were carried out on a Jasco J-820 Spectropolarimeter (Jasco, Tokyo, Japan) with connected a temperature controller (PTC-423L). The CD spectra were obtained by taking the average of three scans in the range of 350–200 nm, with a scan rate of 200 nm/min.

#### Fluorescence spectra

Fluorescence measurements were performed on a Jasco spectrofluorimeter FP-8200 (Jasco, Tokyo, Japan) with 10 nm excitation and 10 nm emission slits and were carried out using 0.4 × 1 cm quartz cuvettes containing 1 ml of solution. Excitation and emission spectra of DNA-AgNCs were recorded in the 200–750 nm range with λ_ex_ = 475 nm/λ_em_ = 560 nm, λ_ex_ = 560 nm/λ_em_ = 610 nm. Sample solution containing 2 μM of fluorescent oligonucleotide in 10 mM Tris-CH_3_COOH buffer (pH 7.5) was equilibrated in a quartz cell at 25 °C for 10 min.

#### Measurements of π-A isotherms and fluorescence spectra at the monolayer interface

To obtain the surface pressure–area (π-A) isotherms for the DODAB (dimethyldioctadecylammonium bromide) monolayer and DODAB/AgNCs complex at the air–water interface we used the computer-contolled balance system (Langmuir trough small, KSV NIMA, Espoo, Finland) as in Ref.^42^. The procedure for creating a monolayer using silver nanoclusters consisted of mixing a DODAB surfactant (5 µL, 1 × 10^–3^ M in CHCl_3_) with a ChONC12 oligonucleotide (5 µL, 1 × 10^–3^ M in water) (1:1 ratio) and AgNO_3_ (6 µl, 1 × 10^–2^ M in water) and such a mixture was applied to the subphase (after 60 min). At a later stage, freshly prepared NaBH_4_ (to reduce Ag^+^ ions) was added behind the barriers. The compression as well as fluorescence measurements of monolayer-silver nanoclusters were carried out analogues as in Ref.^42^. Fluorescence emission spectra for ChONC12-AgNCs were recorded in the 400–750 nm range, with the excitation wavelength of 460 nm and 550 nm (λ_em_ = 550 nm/λ_em_ = 610 nm).

## Data Availability

The data that support the findings of this study are available from the corresponding author upon reasonable request.
